# Molecular Genetic Evidence for the Place of Origin of the Pacific Rat, *Rattus exulans*

**DOI:** 10.1371/journal.pone.0091356

**Published:** 2014-03-17

**Authors:** Vicki Thomson, Ken P. Aplin, Alan Cooper, Susan Hisheh, Hitoshi Suzuki, Ibnu Maryanto, Grace Yap, Stephen C. Donnellan

**Affiliations:** 1 Australian Centre for Ancient DNA, School of Earth and Environmental Sciences, University of Adelaide, Adelaide, South Australia, Australia; 2 Division of Mammals, United States National Museum, Smithsonian Institution, Washington D.C., United States of America, and Department of Archaeology and Natural History, College of Asia and the Pacific, Australian National University, Canberra, Australian Capital Territory, Australia; 3 Austin Academic Centre, The University of Melbourne, Melbourne, Victoria, Australia; 4 Graduate School of Environmental Earth Science, Hokkaido University, Sapporo, Hokkaido, Japan; 5 Zoology Division, Research Centre for Biology, Indonesian Institute of Sciences, Cibinong, Bogor, Indonesia; 6 Environmental Health Institute, National Environment Agency, Singapore, Singapore; 7 South Australian Museum, Adelaide, South Australia, Australia, and Australian Centre for Evolutionary Biology and Biodiversity, University of Adelaide, Adelaide, South Australia, Australia; Institut de Biologia Evolutiva - Universitat Pompeu Fabra, Spain

## Abstract

Commensal plants and animals have long been used to track human migrations, with *Rattus exulans* (the Pacific rat) a common organism for reconstructing Polynesian dispersal in the Pacific. However, with no knowledge of the homeland of *R. exulans*, the place of origin of this human-commensal relationship is unknown. We conducted a mitochondrial DNA phylogeographic survey of *R. exulans* diversity across the potential natural range in mainland and Island Southeast Asia in order to establish the origin of this human-commensal dyad. We also conducted allozyme electrophoresis on samples from ISEA to obtain a perspective on patterns of genetic diversity in this critical region. Finally, we compared molecular genetic evidence with knowledge of prehistoric rodent faunas in mainland and ISEA. We find that ISEA populations of *R. exulans* contain the highest mtDNA lineage diversity including significant haplotype diversity not represented elsewhere in the species range. Within ISEA, the island of Flores in the Lesser Sunda group contains the highest diversity in ISEA (across all loci) and also has a deep fossil record of small mammals that appears to include *R. exulans*. Therefore, in addition to Flores harboring unusual diversity in the form of *Homo floresiensis*, dwarfed stegodons and giant rats, this island appears to be the homeland of *R. exulans*.

## Introduction

Determining the place of origin of commensal animal species is of interest for a number of reasons. First, it is fundamental to understanding the history of commensalism, especially the initial circumstances of development of the relationship, and the timing and directionality of dispersal through human agency. Second, it is an important component of documenting the pattern of natural diversification of the species' broader taxonomic group, which in turn can inform on the historical development of regional biotas. And lastly, knowledge of original habitat associations may help predict the capacity of a species to establish feral populations in new geographic regions and habitats.

**Figure 1:**
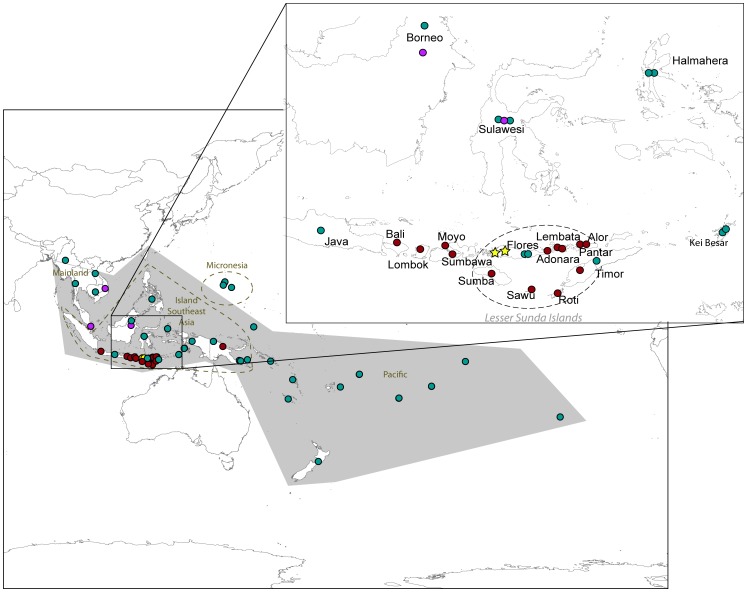
Map of *Rattus exulans* samples used, plus previously published samples used for reference: dark red dots represent the allozyme samples, pink dots represent the *Cytochrome B* samples, green dots represent the modern *CR* samples, and yellow stars represent the ancient *CR* samples. The full geographic range of *R. exulans* is shown in grey, with the Lesser Sunda Islands shown in a dashed oval.

For some species, the history of translocations has been so complex that it can be difficult to pinpoint the natural geographic distribution of the taxon. A case in point is the Pacific Rat (*Rattus exulans*), a commensal murid rodent that is broadly distributed across mainland and island Southeast Asia (MSEA and ISEA, respectively) and also occurs on many of the island groups of both Near and Remote Oceania (Figure 1; and see [1] for further details). Despite this huge geographic range and the historical application of 49 taxonomic names, the species shows limited morphological diversity beyond some variation in body size [2], [3] and it is generally treated as a monotypic species (i.e., lacking subspecies) [1].

While the introduced status of the more remote island populations of *R. exulans* has long been appreciated [4]–[6], no clear view has emerged regarding its place of origin. Musser and Newcomb (ref. [7]: page 524) reviewed information on the pattern of habitat use of *R. exulans* and concluded that it behaved as an introduced taxon across all of ISEA. They speculated that “it may be part of the *Rattus* fauna native to Southeast Asia north of the Isthmus of Kra”. Schwarz and Schwarz [8] thought otherwise and identified Flores in the Lesser Sunda Islands as the place of origin of *R. exulans*, based on the unique occurrence there of white-bellied individuals; and the more general argument that white ventral fur in species of *Rattus* is typical of wild or feral populations, with dark belly fur occurring primarily in commensal settings where there may be strong selection for cryptic colouration. Belly fur in other populations of *R. exulans* is almost always a pale buff colour, with grey bases to the fur.

Phylogeographic studies represent a powerful tool in the investigation of the history of commensal organisms [9], [10]. However, previous phylogeographic studies of *R. exulans* have focused on populations in Near and Remote Oceania [10], [11] and have included minimal coverage of potential source areas (Figure 1). Here we present the results of a broader phylogeographic survey of *R. exulans*, including material from throughout the range of the species in MSEA, from key island groups in ISEA, and from subfossil remains. We use a combination of mitochondrial nucleotide sequences and allozyme frequencies to build a case that Flores in eastern Indonesia is indeed the homeland of *R. exulans*, as suggested by Schwarz and Schwarz [8]. In addition, we investigate the genetic relationship of *R. exulans* to *R. hainaldi*, a morphologically similar species that is endemic to Flores [12] in order to establish the genetic distinctiveness between these congeneric species, which is especially important when dealing with the remains of both species in subfossil cave deposits.

## Materials and Methods

### Ethics Statement

All modern tissue samples were collected prior to formal ethics being required. Collecting permits were obtained from PHKA (Directorate General of Forest Protection and Nature Conservation, Ministry of Forestry, Indonesia). The collecting was done under the auspices of a joint research project between the WA Museum and LIPI (Indonesia Institute of Sciences) with assistance in the field from BKSDA (Nature Conservation Agency, Indonesia). The tissue samples are kept at the South Australian Museum (Adelaide, Australia), Western Australian Museum (Perth, Australia), Hokkaido University (Sapporo, Japan), and National Environment Agency, Singapore (see Table S1 for details). Both ancient tooth samples (ACAD7114 and ACAD7122) were collected from Liang Luar cave on Flores, Indonesia. All necessary permits were obtained for the excavation and collection of the ancient samples, which complied with all relevant regulations. The ancient teeth samples were held at the Australian Centre for Ancient DNA (ACAD) before being completely used up in the DNA extraction procedure.

### Samples

A total of 44 modern tissue samples were sourced from MSEA, ISEA and Micronesia. In addition, we obtained two subfossil rodent incisors from an excavation in Liang Luar cave site on Flores, derived from assemblages associated with uncalibrated radiocarbon dates on wood charcoal of 1241±29 BP and 2011±114 BP, respectively (St Pierre and Aplin, unpublished).

### Modern DNA Extraction, PCR Amplification, and Sequencing

The modern samples were extracted using a Puregene DNA Isolation Kit (Gentra Systems, Minneapolis, Minnesota, USA) following manufacturers protocol for 5–10 mg of fresh or frozen tissue.

PCR reactions for the modern samples were set up using 25 µL volumes containing a final concentration of 1 Unit Immolase DNA Polymerase (Bioline), 2× PCR Buffer (Immolase, Bioline), 7.5 mM MgSO_4_, 1 mM each dNTP, 0.24 µM forward and reverse primers (*cytochrome B*: LM1268 and HM1269; *control region - CR*: EGL-4L and RJ3R; Table S2), and 2–3 µl of template DNA, and performed on an Eppendorf PCR machine using the following conditions: 95**°**C for 10 min, 35 cycles of 94**°**C for 45 s, 55**°**C for 45 s, 72**°**C for 1 min, and a final extension of 6 min at 72**°**C. Amplifications of PCR controls were performed in all experiments to monitor contamination. PCR products were separated by electrophoresis on a 1.5% agarose gel. Successful PCR products (20 µl) were purified using Multiscreen PCR clean-up plates (Millipore Corporation, MA). The purified PCR reactions were sent to the Australian Genome Research Facility for cycle-sequencing in both directions using Big Dye Terminator v3.1 reagents and subsequent capillary sequencing.

### Ancient DNA Extraction, PCR Amplification, and Sequencing

The ancient samples were extracted, PCR amplified and sequenced in specialist aDNA laboratories at the ACAD in Adelaide, South Australia, according to a range of strict protocols and including controls [13].

Each rodent incisor was digested whole and decalcified concurrently with protein digestion by incubation at 55**°**C overnight in 1 mL of extraction buffer (consisting of 0.4725 M EDTA (pH = 8.0), 0.2% sodium dodecyl sulphate (SDS), and 0.7 mg.ml^−1^ Proteinase K). After digestion, samples were then extracted using the DNeasy Kit (Qiagen) following the manufacturer's instructions with any modifications noted. The supernatant was incubated for 10–60 mins at room temperature on a rotary mixer after the addition of the equal volume of AL buffer (Qiagen DNeasy kit) and 0.02 µg.µl^−1^ of carrier RNA. After the incubation and addition of an equal volume of ethanol (100%), the total volume was transferred to a Qiagen DNeasy spin column where it was incubated at room temperature for 10–60 mins. At the elution stage, 100–150 µL of warmed AE buffer was added and then incubated at room temperature for 10–30 mins, before being centrifuged at 8,000 rpm for 1 min; this step was repeated to finish with 200–300 µL of total volume.

PCRs of the ancient material were set up using 25 µL volumes containing a final concentration of 1 Unit Platinum Taq DNA Polymerase High Fidelity (Invitrogen), 1× PCR Buffer (Platinum, Invitrogen), 3 mM MgSO_4_, 200 µM each dNTP, 2 mg.ml^−1^ rabbit serum albumin (Sigma), 1 µM forward and reverse primers, 0.05 Units Shrimp DNase, and 2–3 µl of template DNA. The short fragment primers used to generate the mitochondrial *control region* (*CR*) sequences for the ancient samples are listed in Table S2 (ACAD1526, ACAD1527, ACAD1461, and ACAD1534). PCR reactions for the ancient samples were performed on a Corbett Research Palm Cycler using the following cycling conditions: 94**°**C for 2 min, 55 cycles of 94**°**C for 30 s, 55**°**C for 30 s, 68**°**C for 30 s, and a final extension of 10 min at 68**°**C. Amplifications of extraction and PCR controls were performed in all experiments to monitor contamination. PCR products were separated by electrophoresis on a 3.5% agarose gel. Successful PCR products (20 µl) were purified using AMpure XR magnetic beads (Agencourt). The forward and reverse complements of each fragment were sequenced from the same PCR reaction using the same primers as for the PCR, and Big Dye Terminator v3.1 cycle-sequencing chemistry, followed by purification using CleanSEQ magnetic beads (Agencourt). The sequencing run was conducted on an ABI 3130XC capillary sequencer.

### Sequence analysis

**Figure 2:**
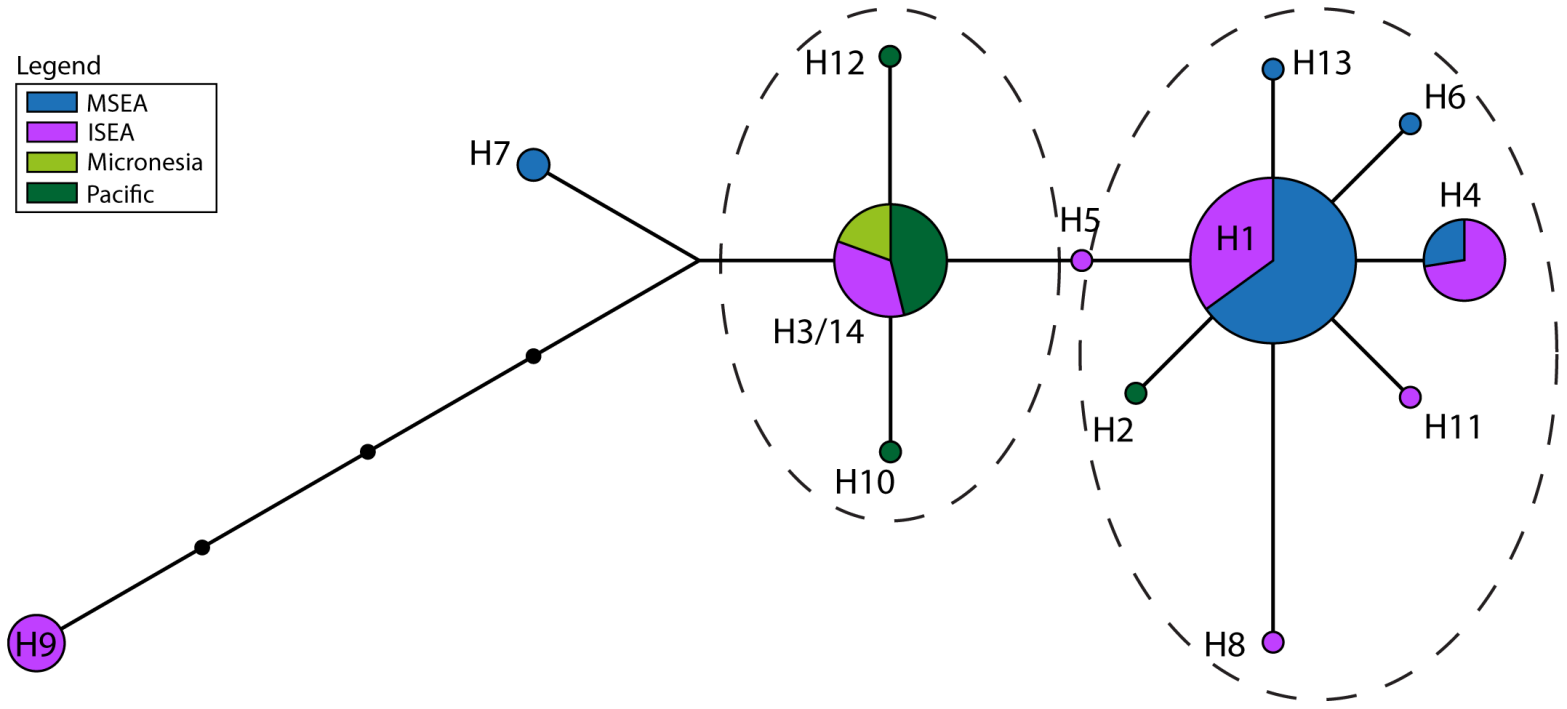
Haplotype network of *Cytochrome B* dataset comprising the common 360 bp across all 89 individuals, and colored by region. The dashed ovals represent the two clusters discussed in the text.

**Figure 3:**
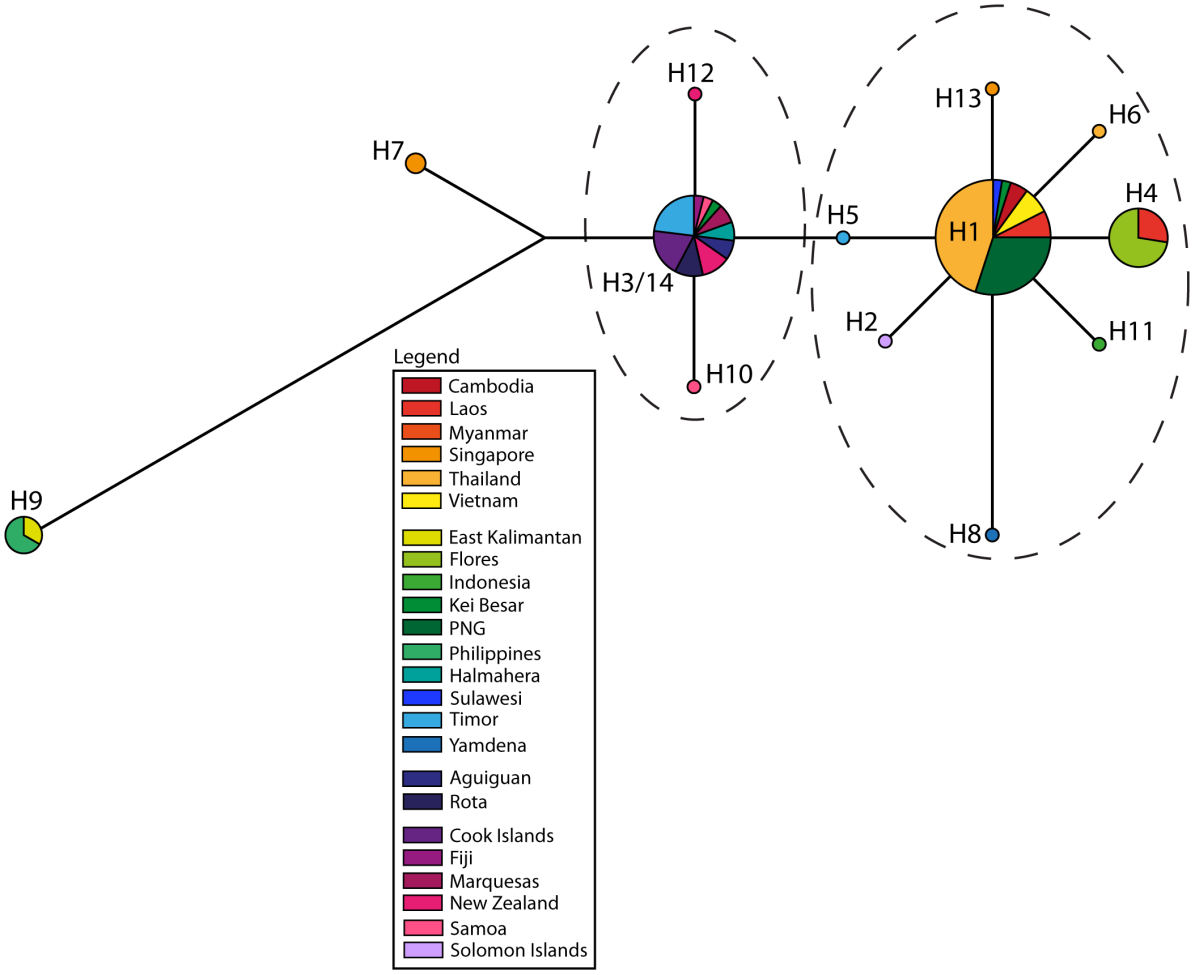
Haplotype network of *Cytochrome B* dataset comprising the common 360 bp across all 89 individuals, and colored by country or island. Samples labeled as ‘Indonesia’ are previously published sequences where the precise provenance is unknown [30]. The dashed ovals represent the two clusters discussed in the text.

**Figure 4:**
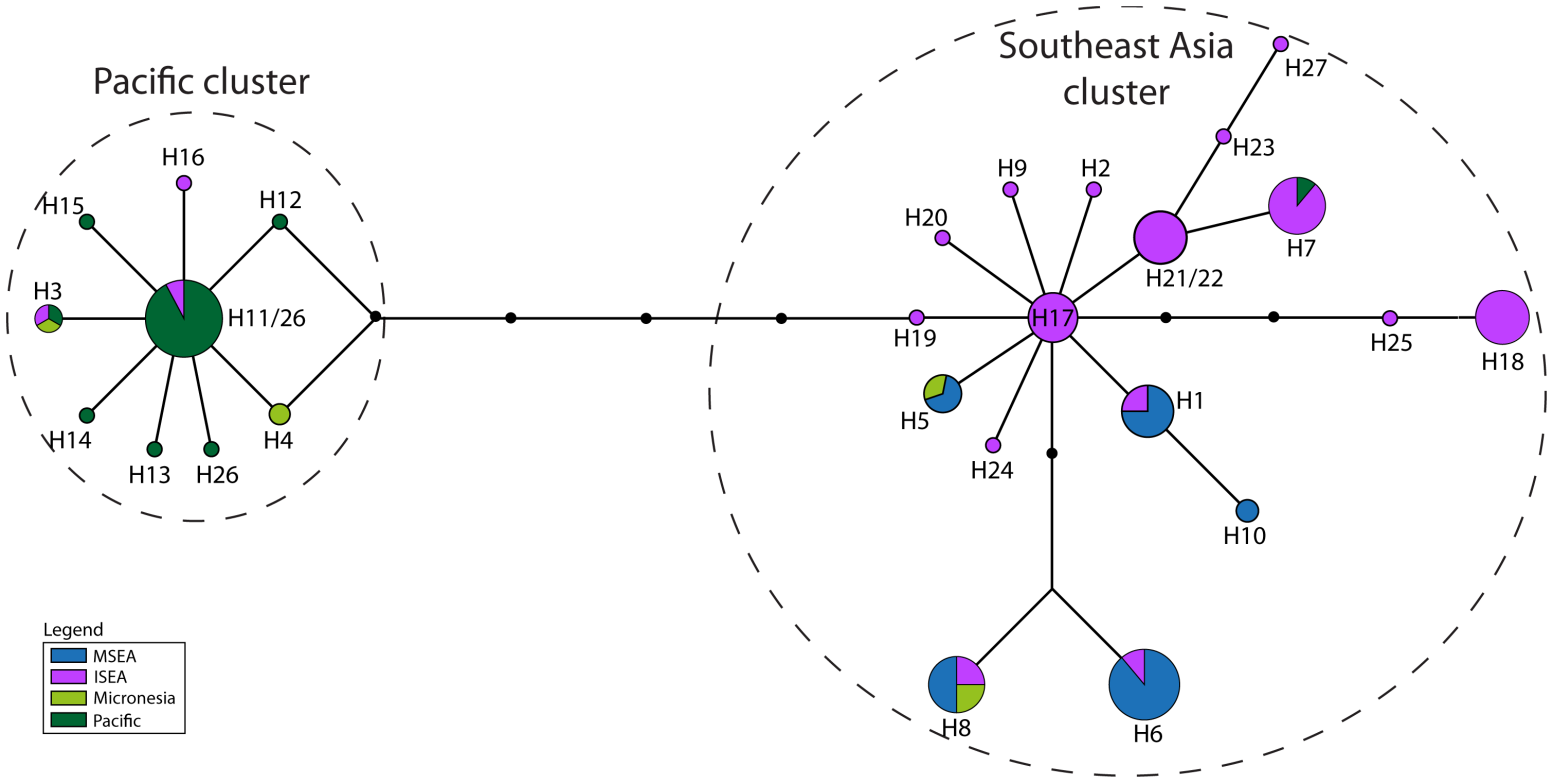
Haplotype network of *Control Region* ‘long’ dataset comprising the common 217 bp across all 72 individuals, and colored by region. The dashed ovals represent the two clusters discussed in the text.

**Figure 5:**
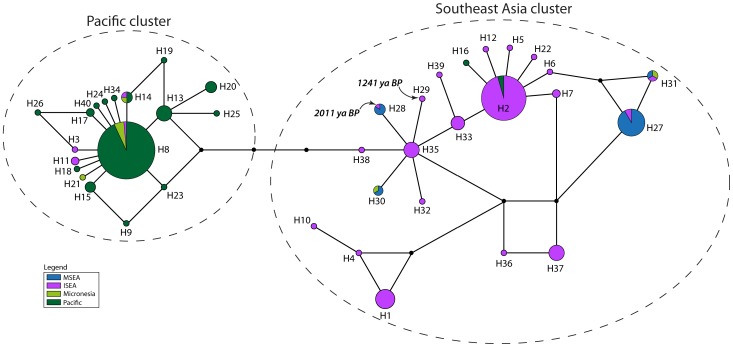
Haplotype network of *Control Region* ‘short’ dataset, comprising the common 92 bp across all 202 individuals, and colored by region. The dashed ovals represent the two clusters discussed in the text.

**Figure 6:**
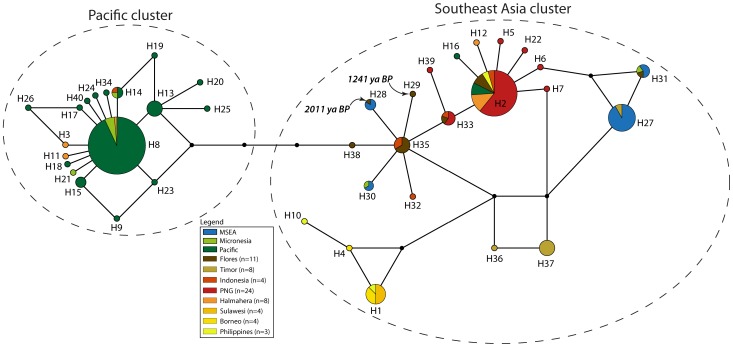
Haplotype network of *Control Region* dataset comprising the common 92 bp across all 202 individuals, and colored by island group. Samples labeled as ‘Indonesia’ are previously published sequences where the precise provenance is unknown [30]. The dashed ovals represent the two clusters discussed in the text.

The forward and reverse sequences were formed into contigs and edited manually in Geneious v5.6.5 [14]. The newly generated sequences were aligned with published sequences using the Muscle alignment algorithm (and refined by eye) to form a *cytochrome B* dataset of 381 bp for 89 sequences, a long *CR* dataset of 544 bp for 72 sequences, and a short *CR* dataset of 107 bp in length for 202 sequences (see Table S1). Population genetic statistics were generated using Arlequin v3.5.1.2 [15]. PhyML v3.0 [16] was used to generate phylogenetic trees with bootstrap support for each dataset, using the best of NNI and SPR tree topology search operations and based on substitution models identified in ModelGenerator v85 [17]. As networks have been shown to be more useful than phylogenetic trees in representing complex evolutionary scenarios [18], we also generated unrooted networks. The long and short *CR* and *cytochrome B* datasets were therefore trimmed to the length common across all sequences (217 bp, 92 bp, and 360 bp respectively) for input into an unrooted median joining network constructed using the Network v4.6.1.1 [19] software (Fluxus Engineering) (Figures 2–6). GenBank accession numbers for all newly generated sequences are KJ155750–KJ155783.

### Allozyme Electrophoresis

Frozen liver samples suitable for allozyme electrophoresis were available from a total of 199 individuals of *R. exulans* and 2 of *R. hainaldi.* Allozyme electrophoresis of liver homogenates was conducted on cellulose acetate gels (‘Cellogel’, Chemetron) according to the methods of Richardson *et al.* [20]. The proteins and enzyme products of 36 presumptive loci were scored (Table S3, see ref. [21] for explanation of locus name abbreviations). Alleles were identified by comparison with samples that were repeatedly included on each gel (internal controls) and through critical side-by-side comparisons (line-ups; see ref. [20]). Alleles were scored numerically in order of increasing electrophoretic mobility (as per ref. [21]).

Gen Alex v6.5 [22] was used to estimate genetic diversity metrics and to perform Principal Coordinates Analysis (PCoA) on genetic distance matrices using codominant genotype distances.

## Results

### Cytochrome B

The highest haplotype diversity was found in the regional population from ISEA (Table 1) and ISEA haplotypes are scattered across the full breadth of the median-joining network (Figures 2–3). Two star-shaped clusters are present, emanating from central haplotypes H1 and H3 but separated by H5. The support values from the phylogenetic tree show bootstrap values separating each star cluster, H1 and H3, from H5 (66% and 63%, respectively; Figure S1). The cluster around H1 mainly comprises haplotypes from MSEA and ISEA, whereas that around H3 contains haplotypes from ISEA and the Pacific (including Micronesia). Regional populations from MSEA and the Pacific show significant values for Tajima's D and Fu's F_S_, indicative of population expansions (Table 1).

**Table 1:** Population genetic summary statistics for the *cytochrome B* dataset (381 bp for 89 sequences).

Group	n	#H	HDiv	NDiv (%)	Ts	Tv	Mean # pairwise diffs (Pi)	Tajima's D	Fu's F_S_
Regional-scale									
ISEA	37	7	0.76	0.59	10	1	2.24	−0.46	0.26
MSEA	32	5	0.38	0.20	5	2	0.76	−1.61*	−1.25
Pacific (incl Micronesia)	20	4	0.28	0.13	3	2	0.50	−1.97*	−1.57
	89								
Island-scale									
MSEA (excl. Thailand)	13	4	0.65	0.40	4	2	1.56	−0.62	0.58
ISEA (excl. Timor and Flores)	22	5	0.58	0.61	8	1	2.31	−0.21	1.27
Remote Pacific	15	4	0.37	0.10	3	2	0.67	−1.91*	−1.22
Timor	7	2	0.29	0.15	2	0	0.57	−1.23	0.86
Thailand	19	2	0.11	0.02	1	0	0.11	−1.16	−0.84
Flores	8	1	0	0	0	0	0	0	0
Micronesia	5	1	0	0	0	0	0	0	0
	89								

ISEA – Island Southeast Asia.

MSEA – Mainland Southeast Asia.

* - Statistically significant p-values (p<0.05 for Tajima's D, p<0.02 for Fu's FS).

#H – Number of haplotypes.

HDiv – Haplotype diversity.

NDiv – Nucleotide diversity.

Ts – Number of transitions.

Tv – Number of transversions.

### Control region (CR)

The *CR* dataset shows higher nucleotide diversity than the *cytochrome B* fragment, and thus contains a potentially richer phylogeographic signal. As found with the *cytochrome B* dataset, the highest haplotype diversity in the *CR*-long dataset was found in the ISEA regional population (Table 2). Within ISEA, the highest genetic diversity was found in Flores (haplotype diversity = 0.89, nucleotide diversity = 0.19%; Table 2). Samples from Timor and the Pacific region gave significant values for Tajima's D and/or Fu's F_S_, indicative of population expansions. The network and phylogenetic tree based on the *CR*-long dataset is widely populated by haplotypes from ISEA (Figure 4 and S2) and features two star clusters, again with both central nodes (H11 and H17) containing haplotypes from ISEA. The network also features a dominant segregation between samples from MSEA and the Pacific region, supported by a bootstrap value of 97% between the two clusters (Figure S2).

**Table 2:** Population genetic summary statistics for the ‘long’ *control region* dataset (544 bp for 72 sequences).

Group	n	#H	HDiv	NDiv (%)	Ts	Tv	Mean # pairwise diffs (Pi)	Tajima's D	Fu's F_S_
Regional-scale									
ISEA	36	13	0.82	0.09	17	2	4.55	0.34	17.82
MSEA	16	5	0.77	0.17	7	1	3.18	1.91	29.62
Pacific (Incl Micronesia)	20	10	0.73	0.01	17	2	2.96	−1.43	−1.32
	72								
Island-scale									
Micronesia	5	4	0.90	0.01	11	2	6.30	0.68	0.86
Flores	12	7	0.89	0.19	8	1	2.84	−0.32	9.95
Indonesia	6	3	0.83	0.03	11	2	6.83	−0.37	3.13
Remote Pacific	15	8	0.59	0.01	14	1	1.58	−2.19*	−1.33
PNG	11	2	0.33	0.01	3	0	0.98	−0.14	7.20
Timor	7	2	0.25	0.02	6	0	1.50	−1.64*	9.48
	72								

ISEA – Island Southeast Asia.

MSEA – Mainland Southeast Asia.

* - Statistically significant p-values (p<0.05 for Tajima's D, p<0.02 for Fu's FS).

#H – Number of haplotypes.

HDiv – Haplotype diversity.

NDiv – Nucleotide diversity.

Ts – Number of transitions.

Tv – Number of transversions.

The network based on the *CR*-short dataset (Figures 5–6) contains more phylogeographic detail for each of the two star clusters, supported by a bootstrap value of 68% (Figure S3). For example, while the cluster around H8 is mainly derived from Pacific populations, it also contains three haplotypes from Halmahera (H8, the central haplotype, and two derived haplotypes that are geographically restricted to Halmahera) and one from Kei Besar in eastern Indonesia (H14). The second major cluster in the *CR*-short network is more complex but comprises haplotypes mainly derived from ISEA and MSEA. Most of the non-terminal haplotypes of this second cluster are found in ISEA (H2, H6, H7, H33, H35, and H38), with Flores containing four of these ancestral haplotypes, plus two other haplotypes that radiate from H35. The central haplotypes were also detected in samples from Papua New Guinea and Indonesia, with Halmahera also yielding one of the common central haplotypes. Among the populations represented by more than 5 individuals, the highest haplotype diversity is observed for the Flores population (Table 3) – nucleotide diversity in the Flores population is twice that of most other populations.

**Table 3:** Population genetic summary statistics for the ‘short’ *control region* dataset (107 bp for 202 sequences).

Group	n	#H	HDiv	NDiv (%)	Ts	Tv	Mean # pairwise diffs (Pi)	Tajima's D	Fu's F_S_
Regional-scale									
ISEA	66	24	0.87	5.30	23	32	4.78	−1.66*	−5.51
MSEA	19	4	0.67	7.32	5	1	2.12	1.58	8.81
Pacific (incl Micronesia)	117	20	0.46	0.93	17	2	1.00	−1.89*	−18.73*
	202								
Island-scale									
Philippines	3	3	1.00	4.67	6	2	5.00	-	0.39
Indonesia	4	4	1.00	4.52	8	1	4.83	−0.15	−0.56
Flores	12	7	0.87	6.25	6	1	1.78	−1.03	0.37
Halmahera	8	5	0.86	3.81	8	1	4.07	1.54	0.35
Micronesia	9	5	0.72	3.04	11	0	3.25	−0.54	0.22
PNG	24	8	0.68	3.76	13	31	3.95	−2.31*	0.65
MSEA	19	4	0.67	7.32	5	1	2.12	1.58	8.81
Borneo	4	2	0.50	0.47	1	0	0.50	−0.61	0.17
Timor	7	3	0.46	0.93	4	0	1.00	−1.53*	0.20
Remote Pacific	108	16	0.44	0.83	13	1	0.89	−1.79*	−13.27
Sulawesi	4	1	0	0	0	0	0	0	-
	202								

ISEA – Island Southeast Asia.

MSEA – Mainland Southeast Asia.

* - Statistically significant p-values (p<0.05 for Tajima's D, p<0.02 for Fu's FS).

#H – Number of haplotypes.

HDiv – Haplotype diversity.

NDiv – Nucleotide diversity.

Ts – Number of transitions.

Tv – Number of transversions.

*CR*-short haplotypes obtained from the two ancient samples from Flores are both derived from H35 (Figures 5–6). Haplotype H28 from the older ancient sample (2011±114 BP) is shared with modern individuals from localities in MSEA, whereas H29 from the younger sample (1241±29 BP) is unique.

### Allozymes

**Figure 7:**
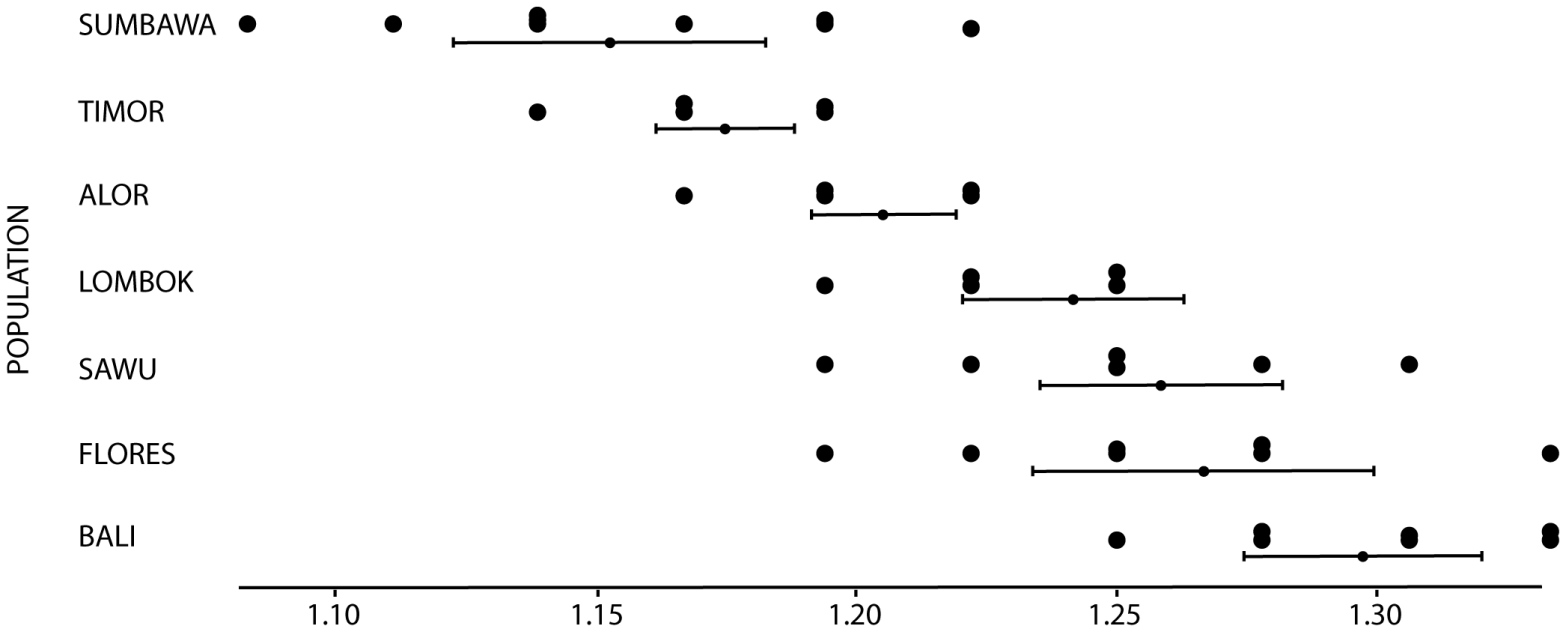
Standardized diversity amongst populations of *R. exulans* using allozyme dataset. Ten randomly selected subsets of 10 individuals were analyzed from each population (with n>10) and assessed for the number of alleles per locus (Na).

The allozyme dataset comprises 36 presumptive loci for 199 individuals of *R. exulans* and 2 of *R. hainaldi* (Tables S3). The Flores population of *R. exulans* contains the highest number of alleles but it also has the greatest sampling density (n = 39 from 7 locations; see Table 4 & Table S3). When the populations are normalized (all populations with >10 individuals had 10 random selections of 10 individuals subsampled for Na), Flores still has amongst the highest number of alleles, exceeded only by Bali (Figure 7 and Table 4).

**Table 4:** Mean number of alleles (Na) per allozyme locus for *R. exulans* per population.

Population	n	Na
Lembata	1	1.028
Pantar	1	1.028
Rota	2	1.056
Moyo	8	1.111
New Guinea	5	1.111
Adonara	1	1.139
Timor	24	1.194
Alor	17	1.222
Sumbawa	35	1.250
Lombok	12	1.278
Java	5	1.306
Sumba	9	1.306
Bali	12	1.333
Sawu	25	1.333
Flores	37	1.389

**Figure 8:**
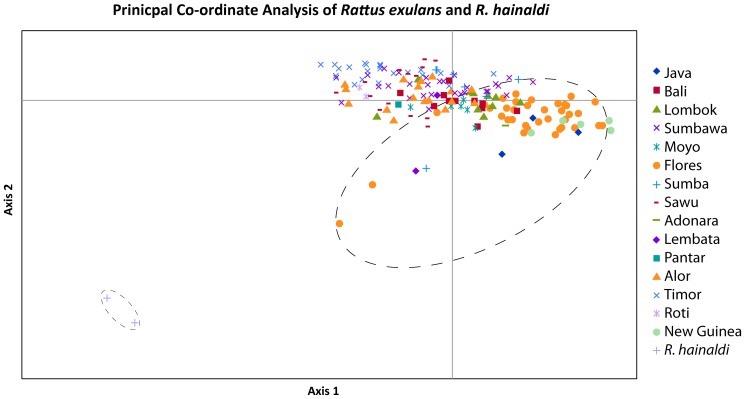
Principal co-ordinate analysis of genetic distances from allozyme variation within *Rattus exulans*, and between *R. exulans* and *R. hainaldi* (two grey crosses outlined with a small ellipse) using co-ordinate 1 on the x-axis and co-ordinate 2 on the y-axis. The large dashed ellipse encompasses the diversity observed in *R. exulans* from Flores.

The PCoA using principal co-ordinates 1 and 2 for *R. exulans* and *R. hainaldi* together illustrates the strong genetic differentiation between the two species (Figure 8). A total of 12 presumptive loci appear to show fixed allelic differences (i.e. no alleles are shared between the two species) although this number may decrease with further sampling of *R. hainaldi* (Table S3).

**Figure 9:**
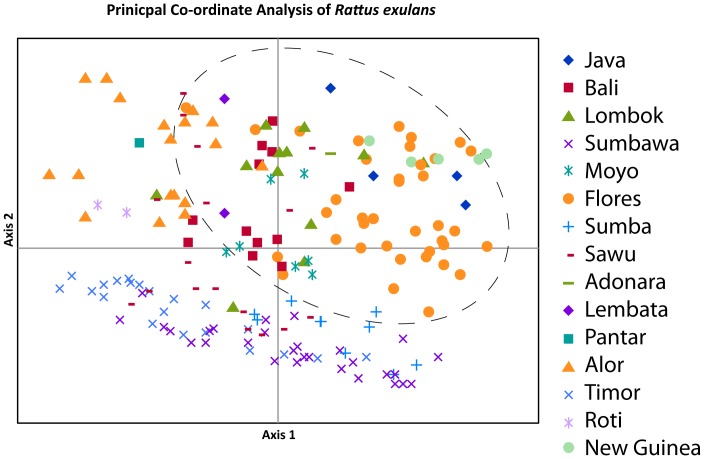
Principal co-ordinate analysis of genetic distances from allozyme variation within *Rattus exulans* using co-ordinate 1 on the x-axis and co-ordinate 2 on the y-axis. The large dashed ellipse encompasses the diversity observed in *R. exulans* from Flores.

The PCoA using principal co-ordinates 1 and 2 on the *R. exulans* samples only (Figure 9) revealed strong geographic structuring of the genetic diversity – the majority of the island populations occupy only small regions of the total PCoA space, with little apparent overlap. By contrast, samples from Flores are widely dispersed across the space, albeit with one major cluster that also includes samples from Java and Papua New Guinea. When principal co-ordinate 1 vs. 3 and principal co-ordinate 2 vs 3 are plotted (Figures S4, S5), the samples from Flores are again widely dispersed across the total PCoA space.

## Discussion

Each of the mtDNA and allozyme datasets contributes important evidence towards narrowing down the place of origin of *R. exulans*. On the basis of the expanded mtDNA dataset, which for the first time includes samples drawn from across the full geographic range of the species, we are confident that *R. exulans* originated in ISEA. Haplotypes from almost all of the major sub-clades are represented in ISEA, including those that are otherwise diagnostic for each of the MSEA and Pacific regions; additionally though, ISEA hosts significant unique haplotype diversity that is not represented elsewhere. While it is possible that ISEA has accumulated haplotype diversity through in-migration from multiple sources, the presence of multiple unique haplotype groups would also require a process of lineage extinction within source areas. A more parsimonious explanation is that the high haplotype diversity found in ISEA is ancestral, with the reduced and largely discrete patterns of diversity seen in each of MSEA and the Pacific being the product of long distance dispersal, with associated founder effects and lineage sorting.

Population expansion analyses conducted on the mtDNA datasets support the notion that ISEA represents a region of long-term residency and relative stability of populations, in contrast with each of MSEA and the Pacific that show strong signals of recent population expansions. The fact that the majority of Pacific samples fall into a star-like cluster supports the notion that dispersal into this region commenced in prehistoric times, thereby providing sufficient time for the generation of local mutations in this region. By contrast, the small number of disconnected haplotypes represented in MSEA suggests a more recent range expansion into this area. Archaeological evidence from the Pacific region confirms an eastward expansion of *R. exulans* commencing around 3500 B.P. [10], [11], [23]. Unfortunately, there is as yet no comparable evidence to date the expansion of *R. exulans* into MSEA.

The allozyme dataset is more restricted geographically but provides a finer resolution insight into genetic patterns within ISEA. Of particular importance, Flores is identified as the island with the highest level of genetic diversity. As with the mtDNA data, several different interpretations of this finding are possible, specifically: 1) the Flores population displays ancestral diversity, with all others being derivatives; 2) the Flores population has been larger and/or more stable and has either preserved or generated more diversity than other islands; and 3) the Flores population is more diverse due to multiple migrations from different source areas. At present it is not possible to choose among these alternatives based on the allozyme data alone. Importantly though, one other possibility – that elevated diversity on Flores is due to introgression following hybridization with the endemic rat *Rattus hainaldi* – can be negated, as none of the alleles that are restricted to Floresian *R. exulans* samples are present in the endemic *R. hainaldi* species.

Given the clear indication of recent range expansion in *R. exulans*, it should be possible to determine its place of origin from the Quaternary fossil record – specifically, as any island or region where the species is represented prior to the mid-Holocene. To date however, no such evidence has been published; instead, *R. exulans* is notable for its absence from numerous fossil-bearing localities of early to late Pleistocene age in each of Indochina [24] and southern China [25], from an early Pleistocene assemblage from Java [26], and from various sites of late Pleistocene to early Holocene age on Sulawesi [27] and Timor [28]. For Flores, published accounts of the 95,000 year Liang Bua sequence indicate that *R. exulans* is restricted to the Upper Holocene levels, while the Pleistocene and Holocene levels contain the similar-sized endemic *R. hainaldi* [29]. However, independent examination of murid specimens from Liang Bua by one of us (KPA) suggests that both species are represented in samples from basal layers of the site. More detailed studies are currently underway to resolve the status of *Rattus exulans* in the fossil record of Flores.

## Supporting Information

Figure S1 **Unrooted Maximum Likelihood phylogenetic tree of** ***Cytochrome B*** **dataset comprising 381 bp across 89 individuals, with bootstrap support values on branches noted.** The dashed ovals represent the two main haplotypes in each cluster discussed in the text.
(TIF)

Figure S2 **Unrooted Maximum Likelihood phylogenetic tree of** ***Control Region*** **‘long’ dataset comprising 544 bp across 72 individuals, with bootstrap support values on branches.** The dashed ovals represent the two main haplotypes in each cluster discussed in the text.
(TIF)

Figure S3 **Unrooted Maximum Likelihood phylogenetic tree of** ***Control Region*** **‘short’ dataset comprising 107 bp across 202 individuals, with bootstrap support values on branches.** The dashed ovals represent the two main haplotypes in each cluster discussed in the text.
(TIF)

Figure S4 **Principal co-ordinate analysis of genetic distances from allozyme variation within** ***Rattus exulans*** **using co-ordinate 1 on the x-axis and co-ordinate 3 on the y-axis.** The large dashed ellipse encompasses the diversity observed in *R. exulans* from Flores.
(TIF)

Figure S5 **Principal co-ordinate analysis of genetic distances from allozyme variation within** ***Rattus exulans*** **using co-ordinate 2 on the x-axis and co-ordinate 3 on the y-axis.** The large dashed ellipse encompasses the diversity observed in *R. exulans* from Flores.
(TIF)

Table S1 **Summary of samples examined in this study.**
(XLSX)

Table S2 **Primer sequences.**
(DOCX)

Table S3 **Allozyme raw data.**
(XLSX)
