# Supplementary figures and images for: Molecular Genetic Evidence for the Place of Origin of the Pacific Rat, Rattus exulans
Source: PLoS One. 2014 Mar 17;9(3):e91356. doi: 10.1371/journal.pone.0091356 (PMC3956674; doi:10.1371/journal.pone.0091356)

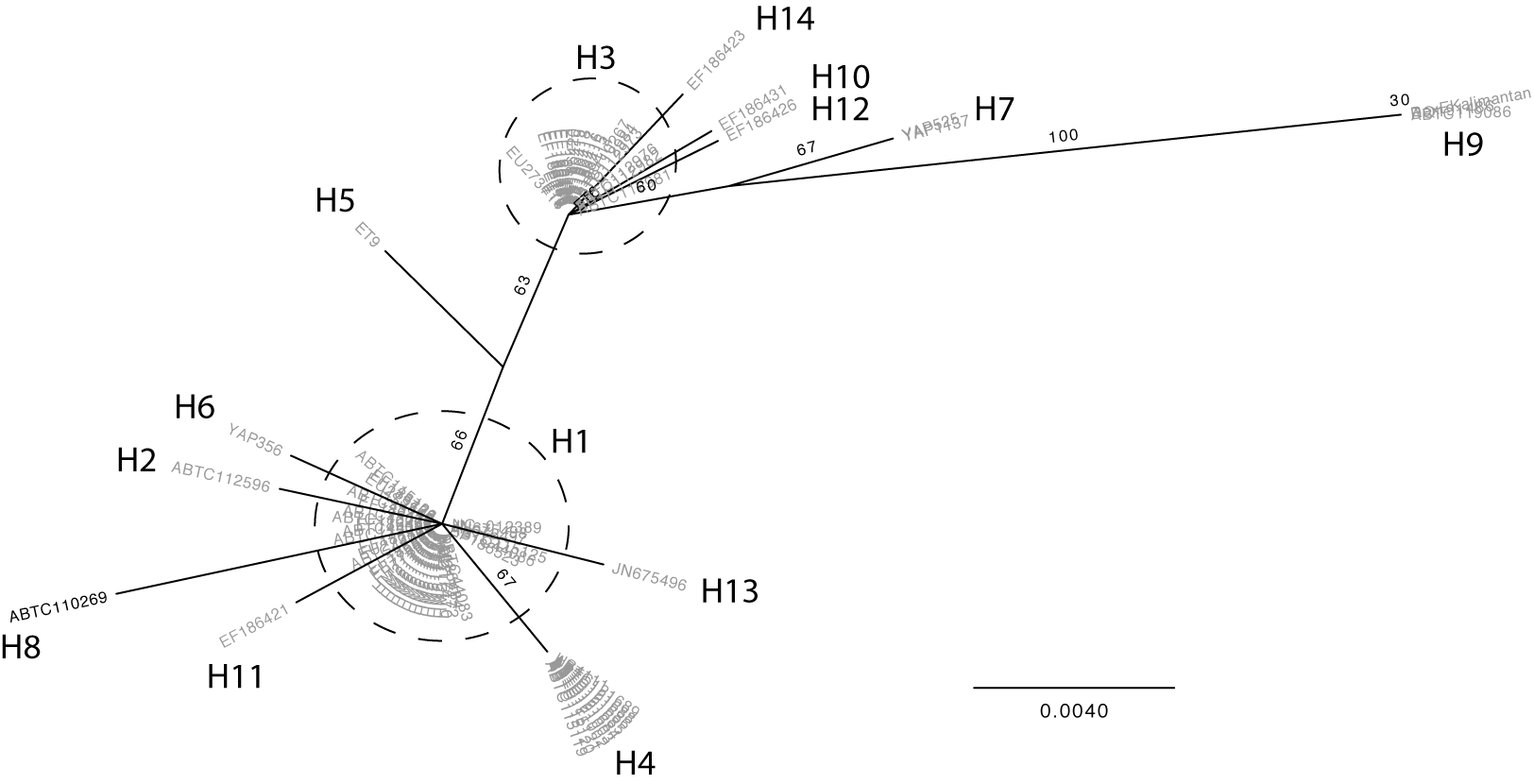

Supplement: Figure S1 — Unrooted Maximum Likelihood phylogenetic tree of Cytochrome B dataset comprising 381 bp across 89 individuals, with bootstrap support values on branches noted. The dashed ovals represent the two main haplotypes in each cluster discussed in the text. (TIF) [file pone.0091356.s001.tif]

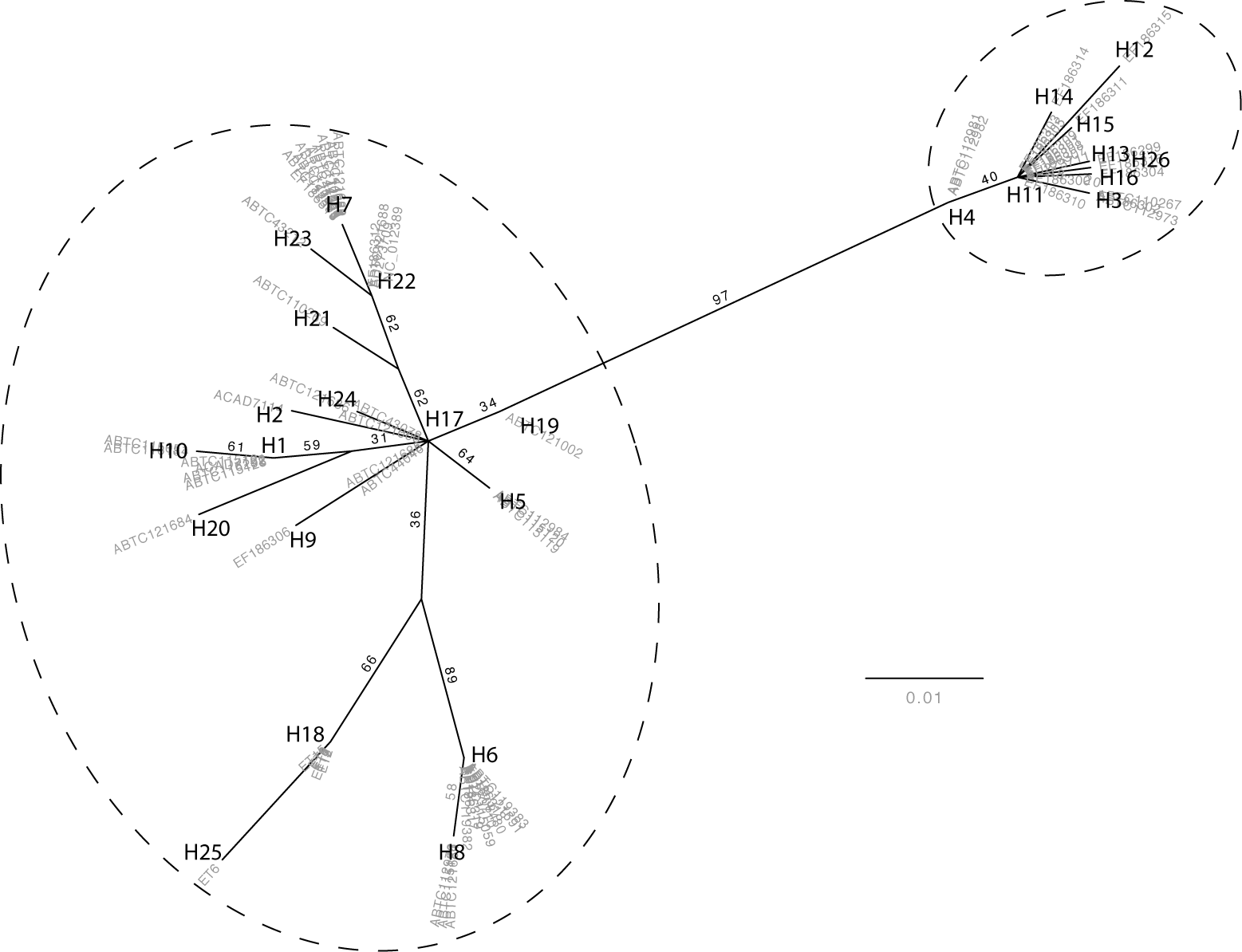

Supplement: Figure S2 — Unrooted Maximum Likelihood phylogenetic tree of Control Region ‘long’ dataset comprising 544 bp across 72 individuals, with bootstrap support values on branches. The dashed ovals represent the two main haplotypes in each cluster discussed in the text. (TIF) [file pone.0091356.s002.tif]

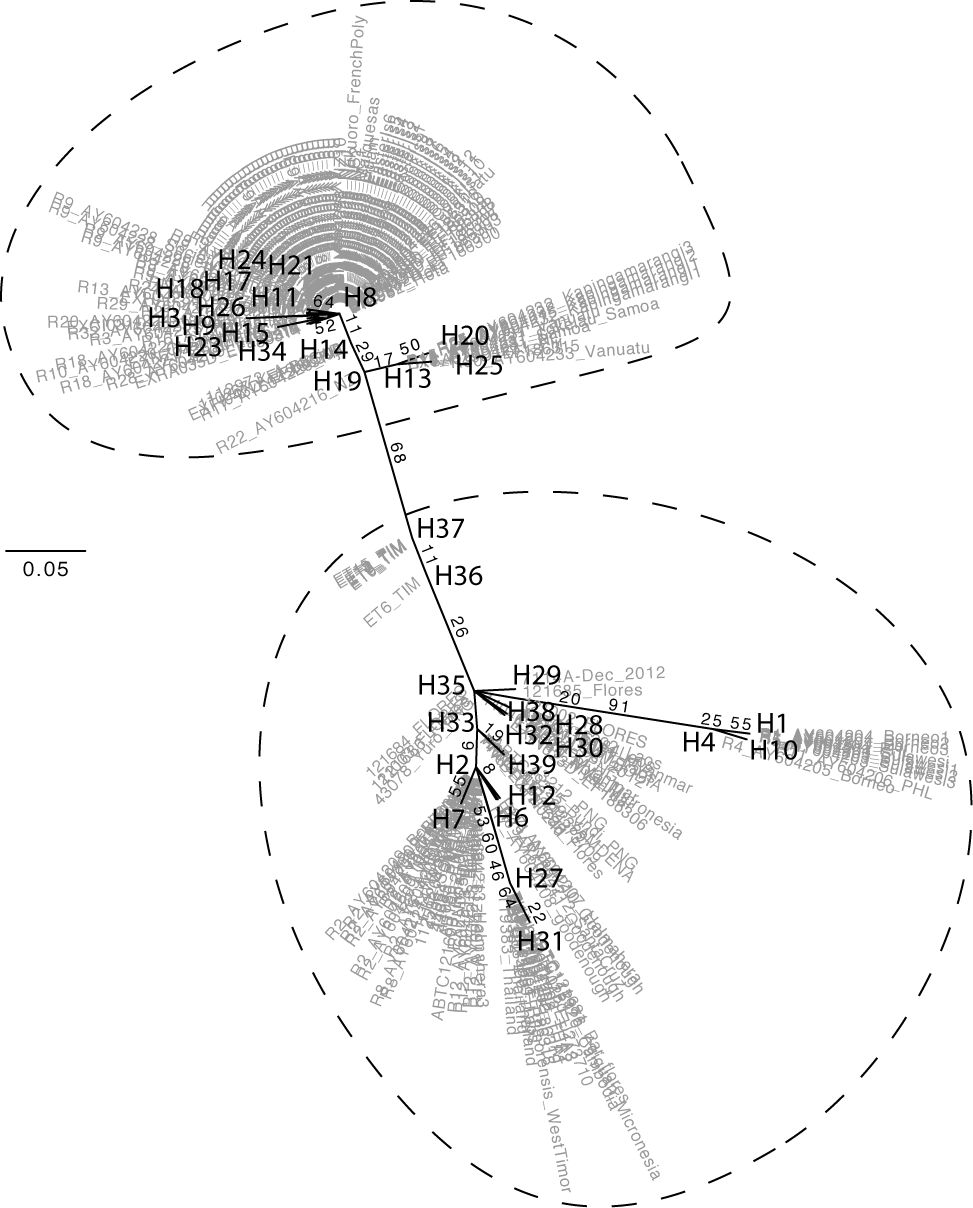

Supplement: Figure S3 — Unrooted Maximum Likelihood phylogenetic tree of Control Region ‘short’ dataset comprising 107 bp across 202 individuals, with bootstrap support values on branches. The dashed ovals represent the two main haplotypes in each cluster discussed in the text. (TIF) [file pone.0091356.s003.tif]

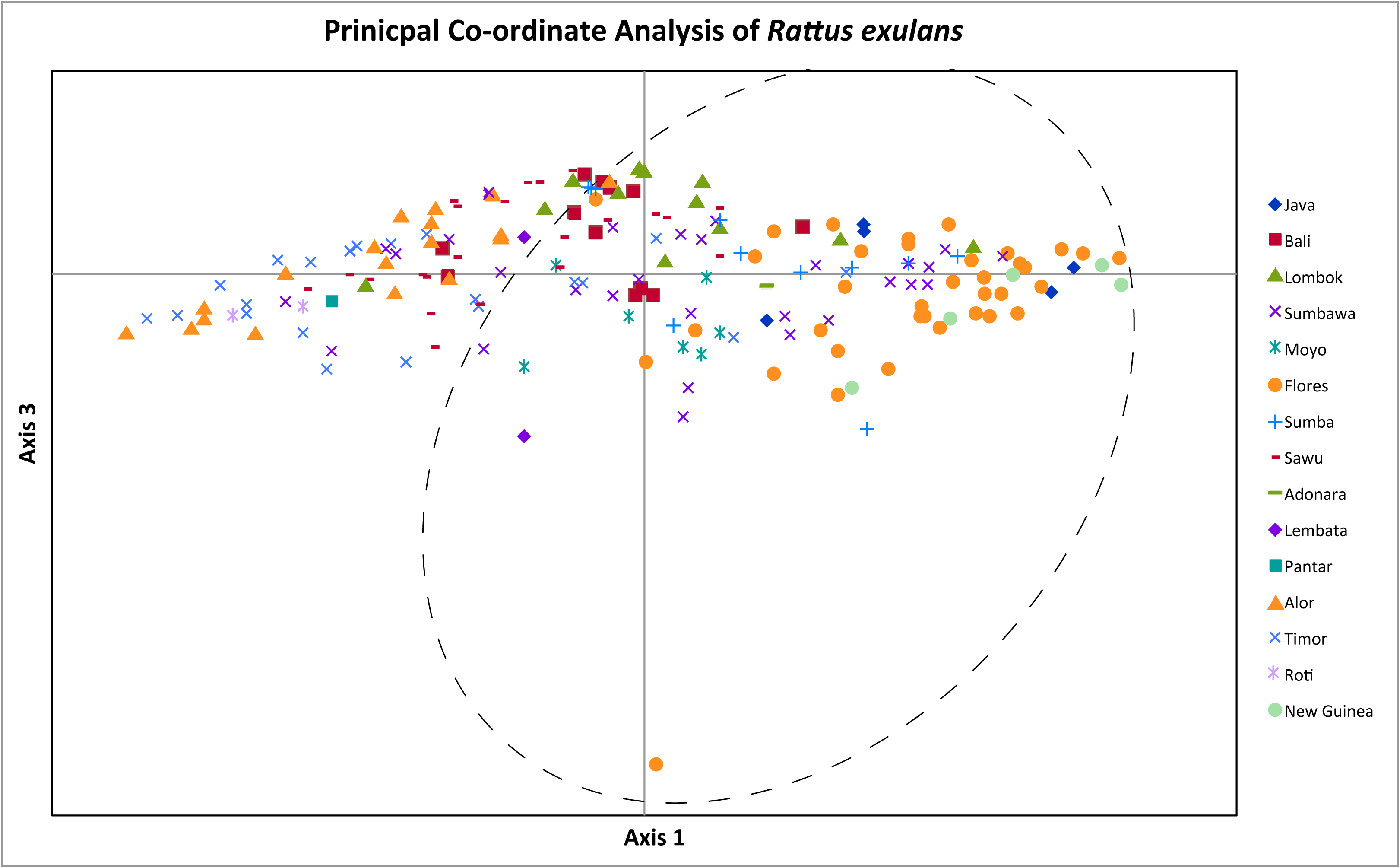

Supplement: Figure S4 — Principal co-ordinate analysis of genetic distances from allozyme variation within Rattus exulans using co-ordinate 1 on the x-axis and co-ordinate 3 on the y-axis. The large dashed ellipse encompasses the diversity observed in R. exulans from Flores. (TIF) [file pone.0091356.s004.tif]

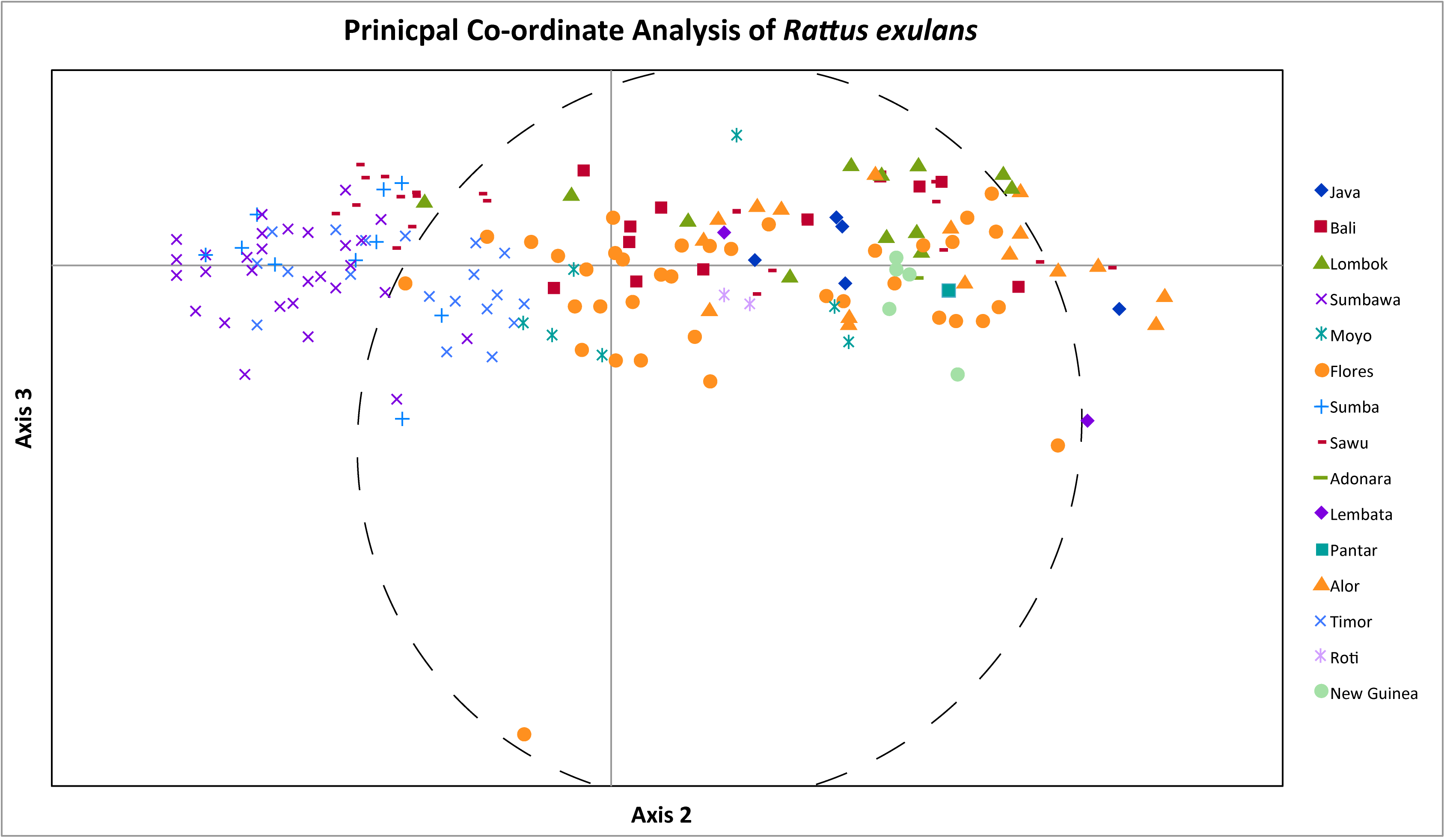

Supplement: Figure S5 — Principal co-ordinate analysis of genetic distances from allozyme variation within Rattus exulans using co-ordinate 2 on the x-axis and co-ordinate 3 on the y-axis. The large dashed ellipse encompasses the diversity observed in R. exulans from Flores. (TIF) [file pone.0091356.s005.tif]
