# Supplementary material for: Molecular Genetic Evidence for the Place of Origin of the Pacific Rat, Rattus exulans
Source: PLoS One. 2014 Mar 17;9(3):e91356. doi: 10.1371/journal.pone.0091356 (PMC3956674; doi:10.1371/journal.pone.0091356)
Supplement: Table S2 — Primer sequences. (DOCX) [file pone.0091356.s007.docx]

Supplementary Information

Table S2. Primer sequences

| Primer name | Region | Primer sequence (5’ to 3’) | Reference |
| --- | --- | --- | --- |
| EGL-4L | *CR* (forward) | CCACCATCAACACCCAAAG | ([24](#_ENREF_24)) |
| RJ3R | *CR* (reverse) | CATGCCTTGACGGCTATGTTG | ([24](#_ENREF_24)) |
| ACAD1526 | *CR* (forward) | CATCTGGTTCTTACTTCAGG | This study |
| ACAD1527 | *CR* (reverse) | GGCATCCGAAAATTAAAAA | This study |
| ACAD1461 | *CR* (forward) | CCCAAGCATATAAGCATGTAA | This study |
| ACAD1534 | *CR* (reverse) | GGATAGTCATATGGAAGAG | This study |
| LM1268 | *Cytochrome B* (forward) | ATTAACCCTCACTAAAGCATGAAAAATCATCGTTGTAA | This study |
| HM1269 | *Cytochrome B* (reverse) | AATACGACTCACTATAGTCTTCATTTTTGGTTTACAAGACCA | This study |
